# Correction: Direct Proof of the *In Vivo* Pathogenic Role of the AChR Autoantibodies from Myasthenia Gravis Patients

**DOI:** 10.1371/journal.pone.0117673

**Published:** 2015-01-30

**Authors:** The PLOS ONE Staff

There are errors in the Funding section. The correct funding information is as follows: This work was funded by the Muscular Dystrophy Association of USA (MDA), the Fight-MG grant of the FP7 EC program, the Association Francaise contre les Myopathies (AFM), the European Commission (Contract No 242210), the Greek GSRT (program “Aristeia”), FP-7 Research potential Program/Regpot SEEDRUG of University of Patras (Grant number: EU FP7 REGPOT CT-2011-285950) and by the Greek national funds through the Operational Program “Education and Lifelong Learning” of the National Strategic Reference Framework (NSRF) Research Funding Program: THALES. The funders had no role in study design, data collection and analysis, decision to publish, or preparation of the manuscript.
