# Correction: Direct Proof of the *In Vivo* Pathogenic Role of the AChR Autoantibodies from Myasthenia Gravis Patients

**DOI:** 10.1371/journal.pone.0120947

**Published:** 2015-03-25

**Authors:** The PLOS ONE Staff

## Notice of Republication

This article was republished on February 12, 2015, to replace Figure 1, which was an erroneous duplication of Figure 2. The publisher apologizes for the error. Please download this article again to view the correct version. The originally published, uncorrected article and the republished, corrected article are provided here for reference.

## Supporting Information

S1 File Originally published, uncorrected article. (PDF)

S2 File Republished, corrected article. (PDF)

## References

[1] Kordas G, Lagoumintzis G, Sideris S, Poulas K, Tzartos SJ (2014) Direct Proof of the *In Vivo* Pathogenic Role of the AChR Autoantibodies from Myasthenia Gravis Patients. PLoS ONE 9(9): e108327 doi: 10.1371/journal.pone.0108327 25259739 10.1371/journal.pone.0108327 PMC4178151

