# Supplementary material for: Correction: Direct Proof of the In Vivo Pathogenic Role of the AChR Autoantibodies from Myasthenia Gravis Patients
Source: PLoS One. 2015 Mar 25;10(3):e0120947. doi: 10.1371/journal.pone.0120947 (PMC4373849; doi:10.1371/journal.pone.0120947)
Supplement: S1 File — (PDF) [file pone.0120947.s001.pdf]

OPEN ACCESS Freely available online

PLOS ONE

Direct Proof of the *In Vivo* Pathogenic Role of the AChR Autoantibodies from Myasthenia Gravis Patients

Gregory Kordas[1], George Lagoumintzis[1], Sotirios Sideris[1], Konstantinos Poulas[1]*, Socrates J. Tzartos[1,2]*

1 Department of Pharmacy, University of Patras, Patras, Greece, **2** Department of Biochemistry, Hellenic Pasteur Institute, Athens, Greece

Abstract

Several studies have suggested that the autoantibodies (autoAbs) against muscle acetylcholine receptor (AChR) of myasthenia gravis (MG) patients are the main pathogenic factor in MG; however, this belief has not yet been confirmed with direct observations. Although animals immunized with AChR or injected with anti-AChR monoclonal Abs, or with crude human MG Ig fractions exhibit MG symptoms, the pathogenic role of isolated anti-AChR autoAbs, and, more importantly, the absence of pathogenic factor(s) in the autoAb-depleted MG sera has not yet been shown by *in vivo* studies. Using recombinant extracellular domains of the human AChR α and β subunits, we have isolated autoAbs from the sera of four MG patients. The ability of these isolated anti-subunit Abs and of the Ab-depleted sera to passively transfer experimental autoimmune MG in Lewis rats was investigated. We found that the isolated anti-subunit Abs were at least as efficient as the corresponding whole sera or whole Ig in causing experimental MG. Abs to both α- and β-subunit were pathogenic although the anti-α-subunit were much more efficient than the anti-β-subunit ones. Interestingly, the autoAb-depleted sera were free of pathogenic activity. The later suggests that the myasthenogenic potency of the studied anti-AChR MG sera is totally due to their anti-AChR autoAbs, and therefore selective elimination of the anti-AChR autoAbs from MG patients may be an efficient therapy for MG.

Citation: Kordas G, Lagoumintzis G, Sideris S, Poulas K, Tzartos SJ (2014) Direct Proof of the *In Vivo* Pathogenic Role of the AChR Autoantibodies from Myasthenia Gravis Patients. PLoS ONE 9(9): e108327. doi:10.1371/journal.pone.0108327

Editor: William Phillips, University of Sydney, Australia

Received May 26, 2014; **Accepted** August 20, 2014; **Published** September 26, 2014

Copyright: © 2014 Kordas et al. This is an open-access article distributed under the terms of the Creative Commons Attribution License, which permits unrestricted use, distribution, and reproduction in any medium, provided the original author and source are credited.

Data Availability: The authors confirm that all data underlying the findings are fully available without restriction. All relevant data are within the paper.

Funding: This work was funded by the Muscular Dystrophy Association of USA (MDA), the Fight-MG grant of the FP7 EC program, the Association Francaise contre les Myopathies (AFM), and by the Greek national funds through the Operational Program "Education and Lifelong Learning" of the National Strategic Reference Framework (NSRF) Research Funding Program: THALES. The funders had no role in study design, data collection and analysis, decision to publish, or preparation of the manuscript.

Competing Interests: The authors have declared that no competing interests exist.

* Email: stzartos@gmail.com (ST); kpoulas@upatras.gr (KP)

Introduction

Myasthenia gravis (MG) is considered the classical organ specific, autoantibody-mediated, and T cell dependent human autoimmune disease [1–6]. MG is associated with autoantibodies (autoAbs) against the acetylcholine receptor (AChR) of the neuromuscular junction (NMJ). These autoAbs are found in approximately 85% of the patients with generalized MG (AChR-MG) [1,2], while the remaining ~15% of MG patients have autoAbs to additional NMJ proteins: MuSK (Muscle-Specific Kinase) [7–9] and LRP4 (lipoprotein receptor-related protein) [10,11] or not yet detectable autoAbs [12]. MG patients are suffering from fatigability and weakness of skeletal muscles, usually due to destruction of the AChRs in the NMJ [2–4,13].

The AChR located in the muscle endplates is a ligand-gated ion channel formed by five homologous subunits with stoichiometry $\alpha_2\beta\gamma\delta$ in the embryos and $\alpha_2\beta\varepsilon\delta$ in the adults. The subunits are arranged symmetrically around a central pore and are embedded in the postsynaptic muscle membrane, with their extracellular domains (ECDs) oriented in the synaptic space. Muscle AChRs bear one binding site per α-subunit for acetylcholine (ACh), the neurotransmitter which is responsible for the transmission of the nerve impulse to the muscle fibers [14,15].

The autoimmune nature of the MG has been proved by several observations supporting the role of anti-AChR autoAbs in the pathogenesis of MG. AChR-MG patients have serum circulating anti-AChR autoAbs, detected also at the NMJ. Moreover, patients' clinical picture improves after plasma or IgG apheresis [16,17]. Transplacental transfer of IgG (including anti-AChR autoAbs) from MG mothers to infants is thought to be responsible for the transient MG observed in the newborns [18,19]. Moreover, the disease can be reproduced in animal models (experimental autoimmune MG, EAMG) either by active immunization with AChR (whole or parts of it) [20–22], or by passive transfer of MG sera [23–25] and monoclonal Abs (mAbs) against the AChR [26,27]. However, although it is clear that animal anti-AChR autoAbs can cause EAMG, it has not yet been directly shown whether the anti-AChR Ab fraction of MG patients' sera is indeed the only or at least the main pathogenic factor in these sera.

The anti-AChR autoAbs in sera of MG patients appear heterogeneous in terms of epitope binding [28]. A large fraction of the autoAbs is directed against a specific region, located at the α-subunits called main immunogenic region (MIR) [29,30]. The MIR is believed to consist of a group of overlapping conformation-dependent epitopes around the 67–76 amino acids of the ECD of the α-subunits, but additional segments also contribute to this antigenic region [31,32]. Its antigenic structure is favored by its position near the synaptic end. In addition to the MIR region, autoAbs against the rest of the α-subunit and the other subunits have also been identified [33,34]. Moreover *E. coli-* and *P.*

pastoris-expressed ECDs of the five human AChR subunits bind anti-AChR autoAbs from various MG sera, underlying the heterogeneity and the variable antigenic specificity of these autoAbs [35].

Epitope variability may explain the lack of correlation between patients' serum autoAb titers and their clinical condition. Indeed, passive transfer experiments with MG sera have demonstrated the induction of the disease only from certain sera [36]. In addition, passive transfer of the disease with mAbs against the AChR has been observed mostly with the use of anti-MIR Abs [26]. In this paper, we isolated autoAbs from the sera of four MG patients using recombinant human α- and β-AChR ECDs. We subsequently tested the ability of the purified autoAbs as well as those of the intact and autoAb-depleted sera to induce EAMG when injected intraperitoneally (*i.p.*) to Lewis rats. Whole sera and purified AChR subunit-specific autoAbs could induce EAMG, whereas the autoAb-depleted sera could not; the latter directly shows that the myasthenic potency of AChR-MG sera is due to their anti-AChR autoAbs. In addition, we showed that the anti-α subunit autoAbs are not the only pathogenic anti-AChR autoAbs though they are much more capable in inducing MG than the anti-β ones.

Materials and Methods

Ethics Statement

Animal experiments were conducted at the Hellenic Pasteur Institute (rat injections, anesthesia and sacrificing) under a protocol approved by the Hellenic Pasteur Institute Animal Care and Use Committee. Anti-AChR auto-Abs isolation from MG sera was performed at the University of Patras. Use of human biological samples was approved by the Bioethics Committee of the Hellenic Pasteur Institute (Permit No. 1288). Written informed consent was taken from all donors participating in this study.

Animals

Lewis rats, 3–4 weeks old, were used. Animals were housed in groups of 4 animals per standard cage in a climate-controlled room on a 12:12 h light:dark cycle with water and standard rodent chow. General anesthesia during animal procedures was provided using Diethyl ether.

MG sera and evaluation of the autoAb titer

Large plasma volumes from MG patients were collected during therapeutic plasmapheresis procedure at the Department of Clinical Therapeutics, Renal Unit, Alexandra Hospital, Athens, Greece. MG serum samples used were from a large number of anti-AChR autoAb-positive sera held in our laboratory at the Hellenic Pasteur Institute for diagnostic purposes as previously described [37]. For simplicity we will use the term "serum" for both plasma and serum. All four patients whose sera were used in this study had generalized mild to moderate MG symptoms (MGFA grades II–III). Interestingly patients MG1 and MG2 (with predominantly anti-α antibodies) had not more severe symptoms than patients MG3 and MG4 (with predominantly anti-β antibodies).

The anti-AChR autoAb titers of the MG sera used in this study were determined by the anti-AChR autoAb assay kit (RSR Ltd, Cardiff, UK) according to the manufacturer's instructions with slight modifications, as previously described [38]. Briefly, the diluted sera or isolated autoAbs [supplemented with NHS (normal human serum) to total 5 μl serum] were incubated overnight at 4°C with 25 μl (about 35,000 cpm) of ^{125}I-AChR solution [^{125}I-α-Bgtx (alpha-bungarotoxin) labeled AChR], and the ^{125}I-AChR-Ab complexes were precipitated by incubation for 2 h at 4°C with 25 μl of goat anti-human IgG serum. The precipitated radioactivity was measured in a γ-counter (2470 WIZARD^2 TM). To test the cross-reactivity of the MG anti-AChR autoAbs with rat AChR, 20 μl of rat muscle extract containing 60 fmol of rat AChR was labeled with approximately 0.1 pmol of ^{125}I-α-Bgtx (~50,000 cpm). After the addition of MG sera or isolated autoAbs (supplemented with NHS to total 5 μl serum), the samples were incubated at 4°C overnight. Antibody-AChR complexes were precipitated by adding 25 μl of goat anti-human IgG serum. The pellets were washed twice with PBS, pH 7.4, 0.5% Triton X-100 and the radioactivity was counted in a γ-counter. Anti-AChR autoAb titers were expressed as nano moles of ^{125}I-α-Bgtx binding sites precipitated per liter of serum (nM).

Competition experiment of serum autoAbs and ^{125}I-α-Bgtx

Human AChR (RSR Ltd, Cardiff, UK) was added to 5 μl serum (mixture of a predetermined quantity of MG1 serum supplemented with NHS to reach the 5 μl). After incubation for 1.5 h, ^{125}I-labeled α-Bgtx (70,000 cpm) was added for 1 h at room temperature. In parallel human AChR was firstly incubated with the ^{125}I-labeled α-Bgtx for 1 h followed by the addition of the MG serum and further incubation for 1.5 h. Complexes were precipitated with goat anti-human serum and the precipitated radioactivity was measured in a γ-counter.

E. coli-expressed ECDs and construction of affinity chromatography CNBr-Sepharose beads columns

Recombinant *E. coli*-expressed human α and β ECDs (α1-210 and β1-221with N-terminal 6×His tag sequence) were produced by growing pET15b/ECD-transformed *BL21(pLysS)DE3* cells as previously described [38]. Purification was performed following the manufacturer's instructions for binding to, and elution from, Ni-NTA (Nickel- Nitrilotriacetic acid) beads (Qiagen). The purified ECDs were used for the construction of CNBr (Cyanogen bromide) -Sepharose columns, in a final concentration 1 mg ECD per mL CNBr-Sepharose according to the manufacturer (Pharmacia-Biotech) guidelines and as previously described [38].

Ig purification by saturated ammonium sulphate

A maximum of 2 ml of MG serum per animal was used for passive transfer experiments. When serum volumes greater than 2 ml had to be used, the Ig of the serum were isolated and concentrated by ammonium sulphate precipitation (40% saturation). When the needed final volume of the precipitated Ig per animal was over 2 ml, it was further concentrated by dialysis membrane covered with polyethelenglycol 40,000.

Affinity purification of the anti-ECD autoAbs

Immunoadsorption experiments were performed by incubating a certain volume of each MG serum or Ig fraction overnight at 4°C with gentle agitation with the appropriate ECD-Sepharose suspension, capable of removing all of the corresponding anti-ECD specific autoAbs (approximately 1 μg of ECD per 2.1 pmol of autoAbs). The reduction in the amount of serum autoAbs represents the fraction of autoAbs in the treated serum reactive with the ECD used. The α- or β-ECD-Sepharose beads with the bound autoAbs were washed three times with PBS and the bound autoAbs were rapidly released from the Sepharose beads by incubation for 5 to 10 seconds at room temperature with 0.2 M glycine-HCl, pH 2.8; then 1/15 volume of 1 M Tris-HCl pH 9.0 was immediately added to the isolated autoAbs to raise the pH,

and the samples were extensively dialyzed against PBS, pH 7.4. Finally, the isolated autoAbs and the depleted sera or Ig fractions were tested for binding to the human and rat AChR by radioimmunoassay (RIA), using ^{125}I-α-Bgtx labeled AChR (see above).

Passive transfer of MG - Clinical assessment

Three to four weeks old Lewis rats were injected *i.p.* with MG serum or serum derivatives, as follows: groups of 3–5 animals were injected with whole serum isolated anti-subunit autoAbs (anti-α or anti-β), depleted sera (without the anti-α or anti-β autoAbs) or with NHS or PBS, as negative controls. Each animal was weighted at the beginning of the experiment and every 24 hours, unless otherwise stated. For clinical examination, rats were evaluated for myasthenic weakness and assigned clinical scores. Briefly, rats were observed on a flat platform for a total of 2 min. They were then exercised by gently dragging them suspended by the tip of the tail across a cage top grid repeatedly (20–30 times) as they attempted to grip the grid. They were then placed on a flat platform for 2 min and again observed for signs of EAMG. Clinical muscle weakness was graded as follows: grade 0, rat with normal posture, muscle strength, and mobility at baseline and after exercise; grade 1, normal at rest but with muscle weakness characteristically shown by a hunchback posture, restricted mobility, and difficulty in raising the head after exercise; grade 2, grade 1 symptoms without exercise during observation period; grade 3, dehydrated and moribund with grade 2 weakness; and grade 4, dead. The evaluator was blinded to treatment status for all clinical evaluations.

Quantification of muscle AChR content

All groups of rats injected with MG sera (MG1–MG4) and their derivatives (autoAb-depleted sera and anti-subunit isolated Abs) as well as the control groups (injected with NHS and PBS) were sacrificed 48 h post-injection. The hind limb muscles of each rat were dissected, weighed and homogenized with a commercial blender using liquid nitrogen to freeze the tissues. The homogenates were then washed twice, using two volumes per muscle net weight (ml/g) of the following buffer: 0.1 M NaCl, 0.01 M Na_2HPO_4-NaH_2PO_4, 0.01 M EDTA, 0.01 M EGTA, 0.001 M PMSF, 0.1 M iodoacetamide, 0.05% NaN_3, 5 U/ml of aprotinin and 0.5 μg/ml of pepstatin, pH 7.4. The pellet was resuspended in an equal volume of 2% Triton X-100 regarding to its weight, in the previous buffer and incubated overnight at 4°C, with gentle agitation. After centrifugation (12,000 g for 30 min) to remove insoluble material, the muscle AChR content was determined as previously described by Lindstrom *et al.,* [39]. In brief, 20 μl aliquots of each muscle extract were labelled with 0.1 pmol of ^{125}I-α-Bgtx and the complexes precipitated using an excess of a mixture of mAbs 192, 195 and 198 followed by the addition of anti-rat IgG, incubation, washing and counting the radioactivity of the pellets. The decrease in the ratio of the Δcpm of samples from autoAb treated animals *versus* Δcpm of samples from control animals was interpreted as the Ab induced AChR-loss.

Confocal microscopy

In order to examine the physical association of MG sera and their derivatives with muscle AChRs at the NMJ, we performed *in vivo* immunofluorescence studies. Twelve hours post-injection groups of rats (2 rats per group) injected with either 0.3 ml MG1 serum or its derivatives (anti-α subunit depleted serum and isolated anti-α subunit Abs), and with Ig from 15 ml of MG3 serum or its derivatives (anti-β subunit depleted serum and isolated anti-β subunit Abs) were sacrificed and the hind limb muscles were isolated from. The hind limb muscle samples were frozen in cold isopentane and stored at −80°C. Ten μm thick sections were taken, allowed to air-dry, and fixed in cold acetone for 10 min. Then the sections were washed twice for 5 min with PBS, and blocked for 25 min with PBS-BSA 3% and FBS 10% (Cappel; MP Biomedicals). After being washed with PBS, the slides were incubated with Alexa Fluor 555-α-Bgtx (1/100) (Invitrogen) for binding on AChR and therefore to identify the NMJ endplates (red) and with F(ab2) donkey anti-human IgG FITC conjugated to identify human Abs (green) for 2 h at room temperature. Then, the slides were washed three times with PBS-Tween 0.1% for 5 min and viewed under a Leica TCS SP5 confocal microscope.

Statistical analysis

AChR loss and rat weight changes were evaluated for statistical significance using a two-tailed unpaired Student's *t*-test, using the Ms Excel 7.0 program.

Results

Selection of MG sera and their Ab specificities

Induction of passive EAMG in rats with obvious clinical symptoms by human MG sera is usually not trivial and requires several injections for several days. In order to achieve MG symptoms by single injections we used young rats (3–4-weeks old), and carefully selected MG sera. In order to select potent MG sera, first we screened over 100 MG sera for binding to both rat and human AChR by RIA. We found that the MG sera showed varying degree of Ab cross-reactivity between the two AChR species (data not shown). For the selection of the most appropriate sera we also took into account their ability to bind to the α- or β-subunit ECDs in order to allow us to isolate and study the corresponding antibody fractions. We finally selected four MG patients' sera (MG1–MG4) of moderate to high titers for rat AChR, for which we had large serum volumes available (Table 1). The selected MG sera were then tested for their autoAbs subunit specificity using immunoadsorbents with α- and β-ECDs. Table 1 shows the characteristics of the four MG sera in respect of autoAb specificities to the human α- or β- AChR subunit ECDs, as well as the cross-reactivity of these MG sera and their derivatives between human and rat AChR. Table 1 shows that their differences in anti-rat AChR titer are smaller than their differences in anti-human AChR titer. Almost half (48.1%) of the MG1 autoAbs are directed against the human α-ECD and are cross-reactive with rat AChR. The remaining anti-AChR Abs (anti-α depleted sera) are not cross-reactive with rat AChR. MG2 and MG3 sera contain autoAbs that bind exclusively α- and β- ECDs, respectively. Both sera (and their isolated autoAbs) were found cross reactive with rat AChR. MG4 serum has the majority of its anti-AChR Abs against the β- ECD (71.2%), while the remaining autoAbs are mainly against the α-ECD. MG4 serum and its derivatives (anti-β Ab depleted serum and isolated anti-β Abs) were all cross-reactive with rat AChR.

Passive transfer by MG sera and their derivatives into rats

We tested the ability of the purified Abs as well as of the intact and autoAb-depleted sera in inducing EAMG when injected *i.p.* to Lewis rats in a range of volumes. Disease severity was assessed on the basis of clinical symptoms and weight change. We initially performed preliminary experiments with the 4 MG sera by which we estimated the approximate volumes of each serum likely to produce clinical symptoms (data not shown). This information was used as a starting point for the below described experiments.

Table 1. Anti-AChR autoAb specificities and autoAb titers of MG sera and their derivatives[a].

MG serum samples	MG serum samples and derivatives	Anti-human AChR Ab titer (nM)	Subunit (ECDs) specificities (% of total anti-human AChR Abs)[b]		Anti-rat AChR Ab titer (nM)
			α subunit	β subunit	
MG1	Whole serum	1150	48.1	2.4	379
	Anti-α Abs depleted serum	596	0	2.4	0
	Isolated anti-α Abs	393	100	0	235
MG2	Whole serum	262	99.8	0	279
	Anti-α Abs depleted serum	0.2	0	0	0
	Isolated anti-α Abs	170	100	0	195
MG3	Whole serum	625	0	98.2	102
	Anti-β Abs depleted serum	0	0	0	0
	Isolated anti-β Abs	455	0	100	63
MG4	Whole serum	29.3	25.3	71.2	23.2
	Anti-β Abs depleted serum	8.4	87.8	0	7.3
	Isolated anti-β Abs	13.9	0	100	12.1

[a]The four MG sera were screened for their Ab specificities for AChR subunits using immunoadsorption ECD columns and the percentages of Ab subunit specificities are shown. The titers of the depleted sera and the isolated Abs are normalized to the corresponding whole serum volumes.
[b]test sera and Ab preparations were incubated with sepharose-immobilised ECDs and the anti-AChR titers of the depleted samples were estimated by RIA with intact AChR.
doi:10.1371/journal.pone.0108327.t001

EAMG by MG1 and its derivatives. MG1 serum had the highest titer for both human and rat AChR among the 4 sera tested. Figure 1A–B show the different volumes of MG1 serum and its derivatives used in the passive transfer experiments, as well as the induced clinical symptoms. A volume of 0.4 ml MG1 whole serum (0.15 nmoles anti-rat AChR Abs) was capable in inducing death to all rats 48 h post-injection. However, Fig. 1A also shows that the anti-α-subunit depleted serum, at even higher volumes (0.4–0.8 ml/animal) was totally inefficient in causing EAMG. The isolated anti-α-subunit Abs (at amounts derived from serum volumes similar with those tested in Fig. 1A) overall were at least similarly efficient with the corresponding volumes of the intact serum (Fig. 1B). Figure 2 depicts the weight change of the group of rats injected with 0.8 ml anti-α depleted MG1 serum 48 h after the injection, while all rats injected with the other MG1 serum fractions had already died. All rats of the group injected with anti-α depleted MG1 serum increased their weight similarly to the control group of rats injected with NHS.

The fact that MG1 serum could induce MG-like symptoms in such small volumes led us to investigate the possibility that the anti-α Abs of MG1 act by blocking the acetylcholine binding site. We carried out a competition experiment between anti-AChR Abs and ^{125}I-α-Bgtx for binding to the AChR. Figure 3 shows that pre-incubation of the AChR with the MG1 serum did not inhibit ^{125}I-α-Bgtx binding to the AChR, suggesting that the MG1 Abs do not interfere with ACh activity.

EAMG by MG2 and its derivatives. Rats injected with 1 and 2 ml MG2 serum or Ig from 5 ml serum did not exhibit any MG-like symptoms (data not shown). On the contrary rats injected with Ig from higher serum volumes (10 ml), equivalent to 2.79 nmoles anti-rat AChR Abs, exhibited mild to moderate EAMG symptoms (Fig. 1C). The body weight of these groups of rats increased less than that of the control groups (Fig. 2). Interestingly the rats that were given purified anti-α Abs from 10 ml serum (1.95 nmoles anti-rat AChR Abs) exhibited severe symptoms (Fig. 1D) and lost dramatically weight (Fig. 2), 48 h post injection. We also injected one rat with anti-α subunit Abs isolated from 20 ml of MG2 serum as shown in Fig. 1D. We found that this rat showed severe MG symptoms and died before the lapse of 48 hours after injection. The anti-α depleted Ab MG fraction, when injected to rats caused no EAMG symptoms.

EAMG by MG3 and its derivatives. MG3 is a high Ab titer serum (625 nM) with autoAbs targeting exclusively the β AChR subunit. The Ig fraction of 4 and 8 ml of whole MG3 serum had no effect. A group of rats were then injected with the Ig fraction of 15 ml of whole MG3 serum corresponding to 1.53 nmoles anti-rat AChR Abs. As shown in Fig. 1E, they showed ambiguous MG symptoms and 48 h after injection they had recovered. The anti-β ECD Abs of 15 ml from MG3 serum were isolated and injected into rats, with none exhibiting any MG symptoms. The isolation of the anti-AChR Abs from the sera, allows using Abs derived from very large serum volumes, which is not possible with crude Ig. We could thus clarify whether MG3 Abs can indeed induce EAMG in rat. Antibodies from 40 ml of whole serum was isolated and injected into each of 2 rats (Fig. 1F), which showed mild MG symptoms within 24 h, while 48 h after injection they fully recovered, but their weight increased less gradually than the control rats (Fig. 2). As also shown in Fig. 1E, 15 ml of anti-β-depleted MG3 serum had no obvious clinical effect when tested in rats and these rats increased their weight normally (Fig. 2).

EAMG by MG4 and its derivatives. MG4 serum had the lowest anti-rat AChR Ab titer (23.2 nM) among the four sera tested in passive transfer experiments. As shown in Fig. 1G, one group of rats was injected with Ig from 5, 10 and 15 ml serum. None exhibited any kind of MG symptoms, and their weight increased similarly with that of the control rats (Fig. 2). In addition, as shown in Fig. 1G, we injected 3 rats and 2 rats with isolated anti-β subunit Abs from 15 ml and 45 ml of MG4 serum each, respectively. No rat showed any MG symptoms.

Muscle AChR content

Using an excess of a mixture of rat AChR specific mAbs, we estimated the content of muscle AChR of each rat group treated with MG sera and derivatives as compared to the control rat group

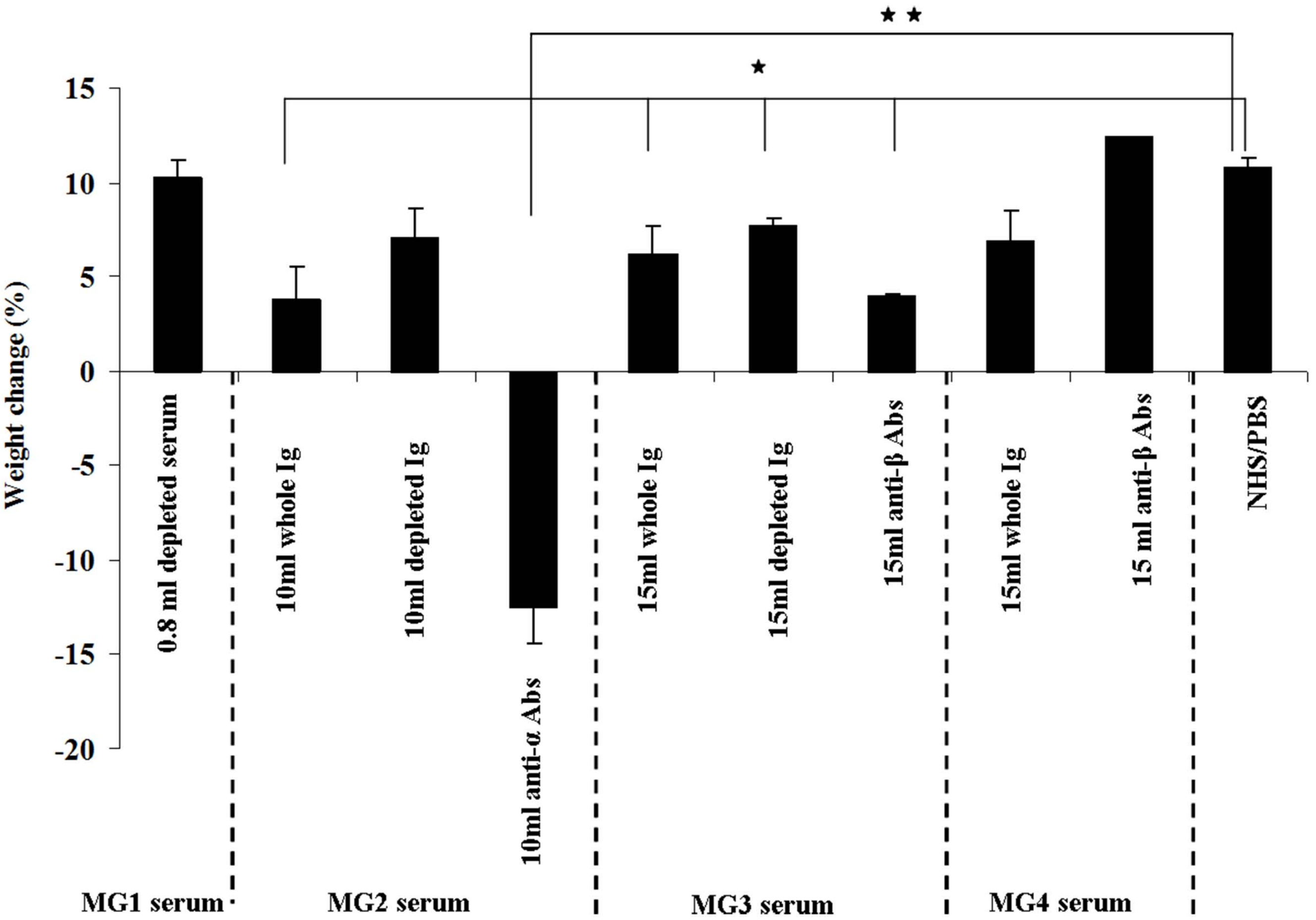

Figure 1. Clinical course of rats 24 and 48 h post-injection with MG sera and their derivatives. The figures show the clinical symptoms of groups of rats injected *i.p.* with the shown volumes of MG sera (or Ig), anti-α/β depleted sera (or Ig) and isolated autoAbs. **A–B:** EAMG-MG1; **C–D:** EAMG-MG2; **E–F:** EAMG-MG3; **G:** (EAMG-MG4). Control groups were injected with NHS and PBS. Disease severity was assessed on the basis of weakness and scored by their ability to grasp, hang and run when provoked: 0–4 (0: no clinical symptoms; 4: dead, see Methods). Points represent mean clinical scores ±SE.
doi:10.1371/journal.pone.0108327.g001

(NHS or PBS) (Fig. 4). The muscle AChR content of the groups of rats injected with PBS are not represented in Fig. 4, as they had similar values to the NHS-treated rats. Administration of 0.25 ml of whole MG1 serum and anti-α subunit Abs isolated from 0.25 ml MG1, significantly reduced (~40%) the AChR molecules at the NMJ of the rats (Fig. 4a). The depleted of the anti-α AChR Abs serum and the control NHS did not cause any effect on rat AChR.

Total MG2 serum reduced the AChR content by about 40% compared to the AChR content of the controls as shown in Fig. 4b. Interestingly, the isolated anti-α Abs from 10 ml of MG2 serum reduced even more drastically the AChR content (~68%), although the actual AChR Ab amount injected to the rats was 30% lower than that of the 10 ml whole serum due to losses during Ab purification. Rats injected with anti-α depleted MG2 serum showed normal levels of AChR concentration.

Ig isolated from 15 ml whole MG3 serum and anti-β subunit Abs isolated from 40 ml MG3 serum both significantly reduced the number of AChRs compared to the anti-β MG3-depleted serum and the controls (NHS and PBS) as it is depicted in Fig. 4c. This confirms the MG symptoms, even though rather weak, induced by this anti-β-subunit serum as shown in Fig. 1E–F.

Whole MG4 serum (15 ml) and anti-β subunit isolated Abs from 45 ml MG4 serum reduced the numbers of AChR (Fig. 4d). Yet, the reduction was the lowest among the 4 MG sera (20–25%) apparently justifying the absence of symptoms in the rats (Fig. 1G).

Overall, the rats in which the antibodies induced <40% AChR loss did not exhibit significant clinical symptoms, whereas in those with >40% AChR loss, disease was observed. The rat group with the highest AChR loss (~68%) in Figure 4 (isolated Abs from MG2) suffered the most severe clinical score.

Detection of IgG deposits at the NMJ by immunofluorescence microscopy

The sections obtained from the rats injected with MG1 whole serum, pure anti-α subunit MG1 Abs, MG3 whole serum and pure anti-β subunit MG3 Abs showed considerable deposition of human IgG at the NMJs (Fig. 5, left and right upper two rows). On the contrary, the depleted MG1 and MG3 sera fractions as well as the control NHS showed little or no deposition at the neuromuscular endplates (Fig. 5, left and right bottom two rows).

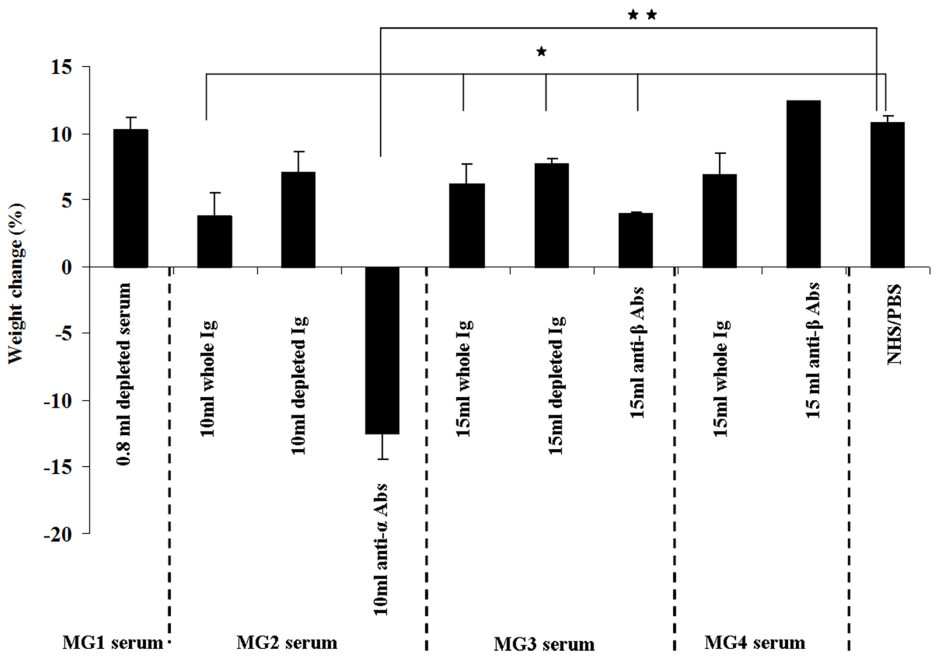

Figure 2. Weight change of rats treated with MG sera and their derivatives. The figures show the percent weight change of groups of rats injected *i.p.* with the shown volumes of MG sera, anti-α/β depleted sera and isolated autoAbs. Control groups were injected with NHS and PBS. Bars represent weight changes (% of controls) +SE, *p<0.05, **p<0.01.
doi:10.1371/journal.pone.0108327.g002

Discussion

MG is an autoimmune disease usually characterized by the presence of autoAbs against the muscle AChR [2–6]. Animals' anti-AChR autoAbs have been clearly shown to cause active or passive EAMG (by immunization with AChR and injection with anti-AChR mAbs). Although MG sera and their isolated whole IgG fractions also induce MG symptoms into animal models, this does not exclude that Abs other than the anti-AChR autoAbs in these sera also contribute to the pathogenicity of the serum or even form the main pathogenic factor. Therefore, direct observation of the pathogenicity of the human MG autoAbs had yet to be achieved. Using recombinant bacterial expressed ECDs of the α and β subunits of human AChR we produced affinity chromatography columns and used them to isolate the anti-α ECD Abs from two MG sera (MG1 and MG2) and the anti-β ECD Abs from another two MG sera (MG3 and MG4). The isolated autoAbs (as well as the whole sera and the Ab-depleted sera) were tested for their ability in inducing EAMG into rats by passive transfer experiments.

Our results showed that the anti-α Abs isolated from MG1 and MG2 sera were capable of inducing severe MG symptoms and death into rats, at least as efficiently as the whole sera. Immunofluorescence studies of the rat hind limb muscles showed deposition of human IgG at muscle endplates, whereas measurements of AChR content showed that the bound anti-AChR autoAbs induced muscle AChR loss, apparently the cause of MG-like symptoms in the rats.

Here, we showed that the anti-α Abs of MG1 could induce severe MG symptoms or death, at approximately 30 times lower amount than anti-α Abs of MG2. This observation prompted us to investigate the possibility that the anti-α Abs of MG1 act by blocking the ACh binding site. We conducted a competition RIA between ^{125}I-α-Bgtx and MG serum and found that the serum autoAbs do not inhibit ^{125}I-α-Bgtx binding to the AChR. This suggests that no autoAbs of MG1 bind near or at the ACh binding site, and therefore their pathogenic effect is probably not due to inhibiting ion channel function.

The strong potency of the MG1 serum in causing EAMG was also tested in mice. Whole MG1 serum was tested in passive transfer experiments in a group of 4 Balb/c mice, 10 weeks old. The critical serum volume that caused death to mice was 0.6 ml, whereas in Lewis rats it was 0.3 ml (data not shown). Thus, although less efficient in mice than in rats (possibly because of

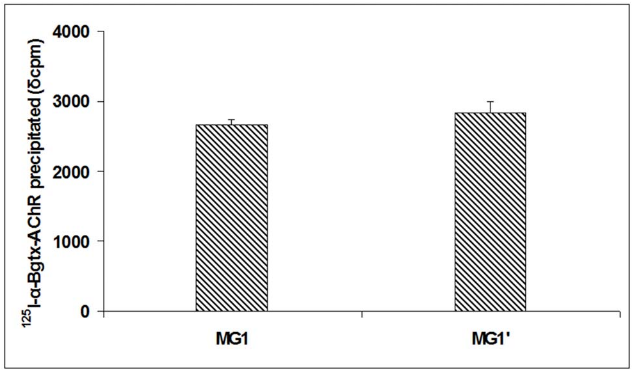

Figure 3. Competition RIA between MG1 autoAbs and ^{125}I-α-Bgtx for binding to the AChR. Anti-AChR Ab precipitation of MG1 serum by RIA and competition RIA for the detection of blocking Abs in the serum. For this, unlabeled AChR was preincubated with MG1 serum followed by the subsequent addition of ^{125}I-α-Bgtx (MG before ^{125}I-α-Bgtx); in parallel AChR was preincubated with ^{125}I-α-Bgtx followed by the subsequent addition of MG1 (MG after ^{125}I-α-Bgtx). The precipitated radioactivity, after the addition of the anti-Ab, was equal in both assays, indicating that no autoAbs of MG1 bind near or at the acetylcholine binding site.
doi:10.1371/journal.pone.0108327.g003

lower cross-reactivity) the strong potency of MG1 is not restricted to the rats.

The anti-β Abs isolated from MG3 and MG4 sera (and MG3 and MG4 total Ig) induced weak or no MG symptoms to animals, respectively. It is possible that the rat complement is not efficiently activated by the human anti-β auto-Abs (of MG3 and MG4) and that the effect on the rats injected with MG3 and MG4 and their derivatives could be mainly due to antigenic modulation of cross-linked AChR. The difference from the anti-α-subunit sera could be due to the presence of two α subunits per AChR molecule which apparently allows the anti-α antibodies to make larger AChR complexes. Since complement fixation is favored by larger antigen-antibody complexes, it would be reasonable for complement to act more strongly with the anti-α antibodies. Moreover, the α-subunits bearing the MIR region are more pathogenic than the other ones in MG patients with more than 50% of the anti-AChR autoabs are directed to the MIR [1,29]. The MIR has been shown to be located on the top of the AChR, favoring cross-linking of AChRs by the anti-MIR antibodies [31,32]. The anti-β auto-Abs in *in vitro* experiments are less capable in cross-linking the AChRs and therefore less capable in causing AChR destruction by both complement and antigenic modulation mechanisms.

This observation is in accordance with our previous studies [37], where we examined the effect of anti-α and anti-β Abs isolated from MG sera *in vitro*, in antigenic modulation experiments. Using TE671 cells we had shown that the anti-α autoAbs are about 4.3 times more effective than the anti-β Abs in internalizing membrane AChR molecules. This can explain the low potency of the anti-β subunit Abs in inducing severe symptoms into rats compared to the anti-α Abs of MG1 and MG2 sera. Nevertheless, it was shown that MG3 Abs did induce mild MG symptoms, weight and AChR loss. Finally, despite the absence of MG symptoms in rats injected with MG4 serum and its isolated anti-β Abs, the muscle AChR concentration was significantly reduced compared to the control groups. Yet, the reduction of about 20% of the total AChR molecules, as expected [40], was not sufficient to affect nerve impulse transduction. Equally important with the induction of EAMG by the isolated anti-α or anti-β Abs, was the observation that none of the depleted fractions caused any MG symptoms to the animals. MG2 and MG3 depleted sera had no anti-AChR autoAbs, as these were adsorbed by the α- and β-ECD columns, respectively. MG1 anti-α depleted serum had about 50% of the total MG1 Abs, but these Abs were not cross-reactive with rat AChR. This suggests that anti-AChR MG autoAbs not only induce EAMG but they seem to be the sole, or at least the main, pathogenic factor in anti-AChR positive MG.

Interestingly, at least in some cases, the isolated Abs seemed more potent than the whole sera. This was more apparent with MG2. The anti-α Abs from the MG2 serum induced clearly stronger clinical symptoms (Fig. 1C–D), weight loss (Fig. 2) and muscle AChR loss (Fig. 4B) than in the rats which were injected with whole MG2 serum Ig. Although this dramatic difference was observed only with MG2 serum, taking into account that the isolated Ab samples had smaller Ab titers from the whole sera or Ig (due to losses during Ab isolation) a trend for higher EAMG efficiency may be noted also for sera MG1 and MG3, overall

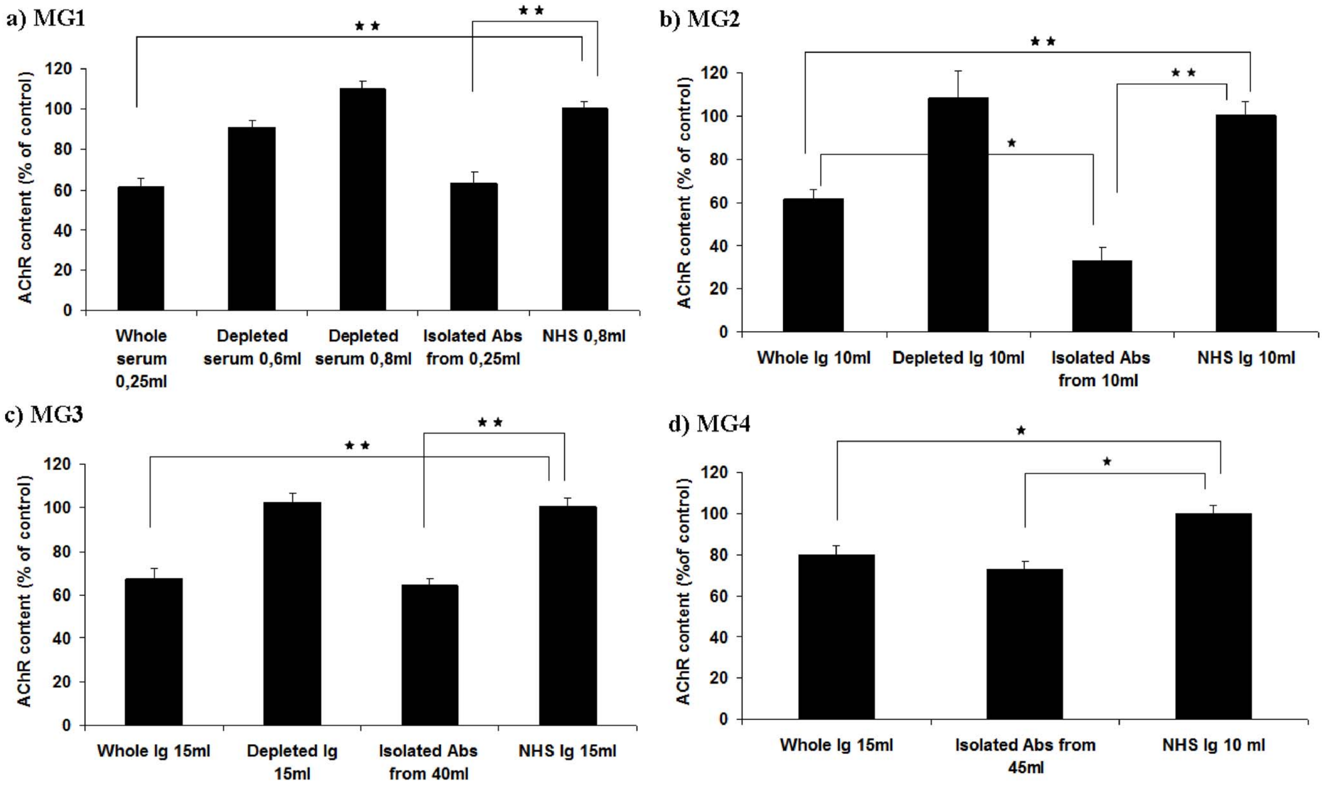

Figure 4. Muscle AChR content of treated rats. The four groups of animals injected with MG sera (MG1–MG4) and their derivatives were sacrificed 48 hours post-injection. The hind limb muscles of each rat were dissected and the extracted AChRs were estimated by RIA. Results are expressed as a percentage of the AChR of the rats receiving NHS only. Results for PBS are not shown but were very similar to the NHS. a, b, c, d: rats injected with sera and derivatives of MG1, MG2, MG3 and MG4 respectively. Bars represent mean AChR content (% of controls) +SE, *$p<0.05$, **$p<0.01$.
doi:10.1371/journal.pone.0108327.g004

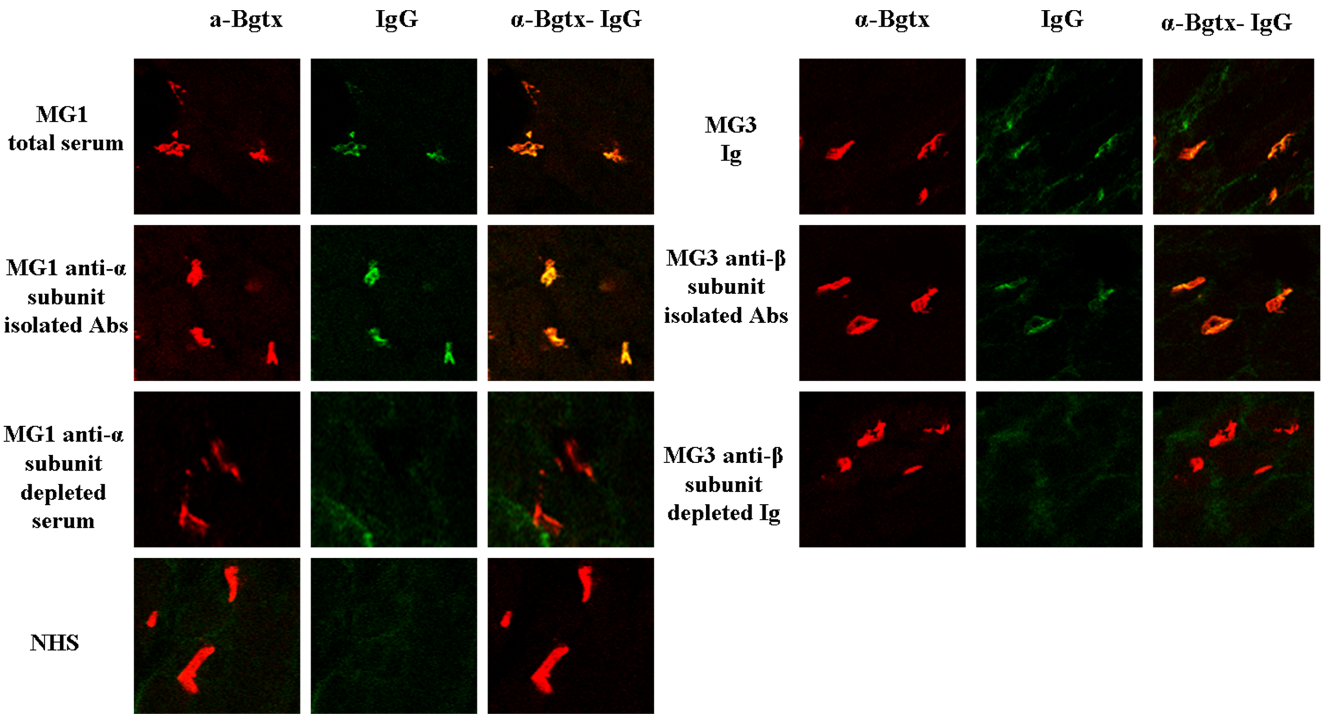

Figure 5. Deposition of IgG at the NMJ of EAMG rats detected by confocal microscopy. Hind limb muscle specimens were obtained from the rats injected with MG1 and MG3 sera and derivatives as well as from rats injected with NHS. Cryostat muscle sections were co-stained with Alexa Fluor 555-α-Bgtx to identify AChR (red) and with F(ab2) donkey anti-human IgG FITC conjugated to identify human autoAbs (green); merge on the right. The immunofluorescence data shown are representative of sections obtained from two rats of each group. Original magnification is 350×.
doi:10.1371/journal.pone.0108327.g005

suggesting the possible presence of protective or suppressive factor(s) in the serum which may partially block the autoAb activity. This observation requires further study in order to identify the potential protective factors in the sera.

Elucidation of the function of anti-AChR autoAbs will help us to reveal their role in the initiation and progression of MG. Furthermore, our results also suggest that the selective removal of the anti-AChR autoAbs from the MG patients, an approach which we are pursuing with ECD-immunoadsorption columns, should be an efficient MG therapeutic approach.

Acknowledgments

The authors wish to thank Mrs. Anna Kokla for excellent technical assistance, Associate Professor Zoi Lygerou for helping with the confocal microscopy experiments, and Dr. Eirini Grapsa for MG plasmas.

Author Contributions

Conceived and designed the experiments: KP SJT. Performed the experiments: GK GL SS. Analyzed the data: GK GL SS KP SJT. Contributed to the writing of the manuscript: GK GL KP SJT.

References

1. Lindstrom JM (2000) Acetylcholine receptors and myasthenia. Muscle Nerve 23: 453–477.
2. Vincent A, Palace J, Hilton-Jones D (2001) Myasthenia gravis. Lancet 357: 2122–2128.
3. Bello-Rivero I, Cervantes M, Torres Y, Ferrero J, Rodríguez E, et al. (2004) Characterization of the immunoreactivity of anti-interferon alpha antibodies in myasthenia gravis patients. Epitope mapping. J Autoimmun 23: 63–73.
4. Infante AJ, Baillargeon J, Kraig E, Lott L, Jackson C, et al. (2003) Evidence of a diverse T cell receptor repertoire for acetylcholine receptor, the autoantigen of myasthenia gravis. J Autoimmun 21: 167–174.
5. Berrih-Aknin S, Frenkian-Cuvelier M, Eymard B (2014) Diagnostic and clinical classification of autoimmune myasthenia gravis. J Autoimmun 48–49: 143–148.
6. Berrih-Aknin S, Le Panse R (2014) Myasthenia gravis: A comprehensive review of immune dysregulation and etiological mechanisms. J Autoimmun 52: 90–100.
7. Marino M, Maiuri MT, Di Sante G, Scuderi F, La Carpia F, et al. (2014) T cell repertoire in DQ5-positive MuSK-positive myasthenia gravis patients. J Autoimmun 52: 113–121.
8. Hoch W, McConville J, Helms S, Newsom-Davis J, Melms A, et al. (2001) Auto-antibodies to the receptor tyrosine kinase MuSK in patients with myasthenia gravis without acetylcholine receptor antibodies. Nat Med 7: 365–368.
9. Hoch W (2003) Molecular dissection of neuromuscular junction formation Trends Neurosci. 26: 335–337.
10. Zhang B, Tzartos JS, Belimezi M, Ragheb S, Bealmear B, et al. (2012) Autoantibodies to lipoprotein-related protein 4 in patients with double-seronegative myasthenia gravis. Arch Neurol 69: 445–451.
11. Zisimopoulou P, Evangelakou P, Tzartos J, Lazaridis K, Zouvelou V, et al. (2014) A comprehensive analysis of the epidemiology and clinical characteristics of anti-LRP4 in myasthenia gravis. J Autoimmun 52: 139–45.
12. Meriggioli MN, Sanders DB (2012) Muscle autoantibodies in myasthenia gravis: beyond diagnosis? Expert Rev Clin Immunol 8: 427–438.
13. Conti-Fine BM, Milani M, Kaminski HJ (2006) Myasthenia gravis: past, present, and future. J Clin Invest 116: 2843–2854.
14. Kalamida D, Poulas K, Avramopoulou V, Fostieri E, Lagoumintzis G, et al. (2007) Muscle and neuronal nicotinic acetylcholine receptors. Structure, function and pathogenicity. FEBS J 274: 3799–3845.
15. Albuquerque EX, Pereira EF, Alkondon M, Rogers SW (2009) Mammalian nicotinic acetylcholine receptors: from structure to function. Physiol Rev 89: 73–120.
16. Newsom-Davis J, Pinching AJ, Vincent A, Wilson SG (1978) Function of circulating antibody to acetylcholine receptor in myasthenia gravis: investigation by plasma exchange. Neurology 28: 266–272.
17. Batocchi AP, Evoli A, Di Schino C, Tonali P (2000) Therapeutic apheresis in myasthenia gravis. Ther Apher 4: 275–279.
18. Tzartos SJ, Efthimiadis A, Morel E, Eymard B, Bach JF (1990) Neonatal myasthenia gravis: antigenic specificities of antibodies in sera from mothers and their infants. Clin Exp Immunol 80: 376–380.
19. Vernet-der Garabedian B, Eymard B, Bach JF, Morel E (1989) Alpha-bungarotoxin blocking antibodies in neonatal myasthenia gravis: frequency and selectivity. J Neuroimmunol 21: 41–47.

20. Patrick J, Lindstrom J (1973) Autoimmune response to acetylcholine receptor. Science 180: 871–872.
21. Christadoss P, Poussin M, Deng C (2000) Animal models of myasthenia gravis. Clin Immunol 94: 75–87.
22. De Baets M, Stassen MH (2002) The role of antibodies in myasthenia gravis. J Neurol Sci 202: 5–11.
23. Toyka KV, Brachman DB, Pestronk A, Kao I (1975) Myasthenia gravis: passive transfer from man to mouse. Science 190: 397–399.
24. Toyka KV, Drachman DB, Griffin DE, Pestronk A, Winkelstein JA, et al. (1977) Myasthenia gravis. Study of humoral immune mechanisms by passive transfer to mice. N Engl J Med 296: 125–131.
25. Buschman E, van Oers N, Katz M, Murgita RA (1987) Experimental myasthenia gravis induced in mice by passive transfer of human myasthenic immunoglobulin. Evidence for an ameliorating effect by alpha-fetoprotein. J Neuroimmunol 13: 315–330.
26. Tzartos S, Hochschwender S, Vasquez P, Lindstrom J (1987) Passive transfer of experimental autoimmune myasthenia gravis by monoclonal antibodies to the main immunogenic region of the acetylcholine receptor. J Neuroimmunol 15: 185–194.
27. Tzartos SJ (1988) Myasthenia gravis studied by monoclonal antibodies to the acetylcholine receptor. In Vivo 2: 105–110.
28. Berrih-Aknin S (1995) Myasthenia gravis, a model of organ-specific autoimmune disease. J Autoimmun 8: 139–143.
29. Tzartos SJ, Barkas T, Cung MT, Mamalaki A, Marraud M, et al. (1998) Anatomy of the antigenic structure of a large membrane autoantigen, the muscle-type nicotinic acetylcholine receptor. Immunol Rev 163: 89–120.
30. Lindstrom J, Luo J, Kuryatov A (2008) Myasthenia gravis and the tops and bottoms of AChRs: antigenic structure of the MIR and specific immunosuppression of EAMG using AChR cytoplasmic domains. Ann N Y Acad Sci 1132: 29–41.
31. Lindstrom J, Luo J (2012) Myasthenogenicity of the main immunogenic region. Ann N Y Acad Sci 1274: 9–13.
32. Beroukhim R, Unwin N (1995) Three-dimensional location of the main immunogenic region of the acetylcholine receptor. Neuron 15(2): 323–331.
33. Tzartos SJ, Seybold ME, Lindstrom JM (1982) Specificities of antibodies to acetylcholine receptors in sera from myasthenia gravis patients measured by monoclonal antibodies. Proc Natl Acad Sci U S A 79: 188–192.
34. Ragheb S, Mohamed M, Lisak RP (2005) Myasthenia gravis patients, but not healthy subjects, recognize epitopes that are unique to the epsilon-subunit of the acetylcholine receptor. J Neuroimmunol 159: 137–145.
35. Kostelidou K, Trakas N, Tzartos SJ (2007) Extracellular domains of the beta, gamma and epsilon subunits of the human acetylcholine receptor as immunoadsorbents for myasthenic autoantibodies: a combination of immunoadsorbents results in increased efficiency. J Neuroimmunol 190: 44–52.
36. Mossman S, Vincent A, Newsom-Davis J (1988) Passive transfer of myasthenia gravis by immunoglobulins: lack of correlation between AChR with antibody bound, acetylcholine receptor loss and transmission defect. J Neurol Sci 84: 15–28.
37. Sideris S, Lagoumintzis G, Kordas G, Kostelidou K, Sotiriadis A, et al. (2007) Isolation and functional characterization of anti-acetylcholine receptor subunit-specific autoantibodies from myasthenic patients: receptor loss in cell culture. J Neuroimmunol 189: 111–117.
38. Zisimopoulou P, Lagoumintzis G, Poulas K, Tzartos SJ (2008) Antigen-specific apheresis of human anti-acetylcholine receptor autoantibodies from myasthenia gravis patients' sera using Escherichia coli-expressed receptor domains. J Neuroimmunol 200: 133–141.
39. Lindstrom JM, Einarson BL, Lennon VA, Seybold ME (1976) Pathological mechanisms in experimental autoimmune myasthenia gravis. Immunogenicity of syngeneic muscle acetylcholine receptor and quantitative extraction of receptor and antibody-receptor complexes from muscles of rats with experimental automimmune myasthenia gravis. J Exp Med 144: 726–738.
40. Christadoss P, Lindstrom J, Munro S, Talal N (1985) Muscle acetylcholine receptor loss in murine experimental autoimmune myasthenia gravis: correlated with cellular, humoral and clinical responses. J Neuroimmunol 8: 29–41.
